# Mortality Predictors in Patients Diagnosed with COVID-19 in the Emergency Department: ECG, Laboratory and CT

**DOI:** 10.3390/medicina57060629

**Published:** 2021-06-17

**Authors:** Aslı Türkay Kunt, Nalan Kozaci, Ebru Torun

**Affiliations:** 1 Department of Emergency Medicine, Faculty of Medicine, Alanya Alaaddin Keykubat University, Antalya 07450, Turkey; nalankozaci@gmail.com; 2 Department of Radiology, Faculty of Medicine, Alanya Alaaddin Keykubat University, Antalya 07450, Turkey; drebrutorun@yahoo.com

**Keywords:** computerized tomography, COVID-19, emergency medicine, laboratory, mortality

## Abstract

*Background and Objectives*: The aim of this study was to investigate parameters that can be used to predict mortality in patients diagnosed with COVID-19 in the emergency department (ED). *Materials and Methods*: Patients diagnosed with COVID-19 in the ED were included in this prospective study. The patients were divided into two groups. The surviving patients were included in Group 1 (survivors), and the patients who died were included in Group 2 (non-survivors). The electrocardiogram (ECG), laboratory results and chest computerized tomography (CCT) findings of the two groups were compared. The CCT images were classified according to the findings as normal, mild, moderate and severe. *Results*: Of the 419 patients included in the study, 347 (83%) survived (survivor) and 72 (17%) died (non-survivor). The heart rate and respiratory rate were found to be higher, and the peripheral oxygen saturation (SpO_2_) and diastolic blood pressure (DBP) were found to be lower in the non-survivor patients. QRS and corrected QT interval (QTc) were measured as longer in the non-survivor patients. In the CCT images, 79.2% of the non-survivor patients had severe findings, while 11.5% of the survivor patients had severe findings. WBC, neutrophil, NLR, lactate, D-dimer, fibrinogen, C- Reactive Protein (CRP), urea, creatinine, creatine kinase-MB (CK-MB) and hs-Troponin I levels were found to be higher and partial pressure of carbon dioxide (PCO_2)_, base excess (BE), bicarbonate (HCO_3)_, lymphocyte eosinophil levels were found to be lower in non-survivor patients. The highest AUC was calculated at the SpO_2_ level and the eosinophil level. *Conclusions*: COVID-19 is a fatal disease whose mortality risk can be estimated when the clinical, laboratory and imaging studies of the patients are evaluated together in the ED. SpO_2_ that is measured before starting oxygen therapy, the eosinophil levels and the CT findings are all important predictors of mortality risk.

## 1. Introduction

In December 2019, a new coronavirus disease was identified in Wuhan, China, which quickly spread to become a global pandemic. Studies have shown that the disease severity is variable [1,2,3]. In mild cases of COVID-19, the usual symptoms of the respiratory tract similar to other respiratory viruses are observed, while in critical cases, severe pneumonia, acute respiratory distress syndrome (ARDS), multiple organ failure and death [4] can occur. Therefore, early diagnosis, severity analysis and treatment of COVID-19 patients are important for reducing morbidity and mortality, as well as for controlling and preventing the epidemic. Many studies have been conducted reporting the clinical characteristics of COVID-19 patients. The impact of patients’ comorbid diseases, along with electrocardiography (ECG), laboratory and imaging results, were investigated in these studies. However, critically ill patients are generally admitted to emergency departments (ED), and laboratory and imaging studies in the ED are limited and time-consuming. For this reason, easily accessible and simple markers showing the mortality risk of COVID-19 patients are needed. However, the number of reports describing the clinical features of serious or critical COVID-19 patients is low. The aim of this study was to investigate the parameters that can be used to predict mortality in patients diagnosed with COVID-19 in the ED.

## 2. Materials and Methods

### 2.1. Study Design and Setting

This prospective study was initiated after approval by the Alanya Alaaddin Keykubat University (ALKU) Faculty of Medicine Ethics Committee. The single-center, observational study was conducted in the ED of the ALKU Alanya Training and Research Hospital. Since this hospital is located in a tourist area, tourists from many different countries are examined there. In addition, it is the only hospital treating COVID-19 patients in the region. Of the daily average of 400 patient admissions to the ED, approximately 40 had a preliminary diagnosis of COVID-19 during 15 June 2020 and January 2021. Patients aged 18 and over who were diagnosed with COVID-19 in the ED between 15 June 2020 and 15 January 2021 were included in the study.

The diagnoses of COVID-19 in the patients were confirmed with reverse transcription polymerase chain reaction (PCR) tests, which were performed on nasopharyngeal swab samples. Samples for the PCR testing of the patients were taken in the ED. Patients younger than 18 years of age, pregnant women and patients with incomplete data; patients who did not have chest computerized tomography (CCT) imaging or had imaging conducted at an external center; patients who had cysts, masses, pneumothorax, hydrothorax, pleural effusion, pericardial effusion or an aortic aneurysm on the CCT images; and patients who were diagnosed with COVID-19 and treated outside the ED were not included in the study. A standard data collection form was created for the study. The file numbers, age (in years), gender, history, vital signs at the time of emergency admission, electrocardiography (ECG) and laboratory results, CCT reports, hospital admission and clinical outcomes of the patients were recorded on these forms. Informed consent forms were obtained from all patients included in the study. The informed consent form was not obtained from patients who were referred from the hospital and were not accompanied by their relatives. Patients’ outcomes were followed up from the Turkish Ministry of Health’s Death Report System and hospital’s database retrospectively, and patients were classified as either an in-hospital death or discharged from the hospital according to these results.

### 2.2. Evaluation of the Electrocardiogram

At admission to the emergency department, 12 derivation ECGs were taken from each patient and interpreted by the emergency medicine instructor. In ECG analysis, a standardized reading protocol was used to evaluate ECG intervals, speed, rhythm, axis, QRS morphology, voltage and ST or T wave abnormalities. The corrected QT interval (QTc) was calculated according to the Bazett formula.

### 2.3. Biochemical Analysis

The reference values reported in healthy adults were as follows: 0–0.5 mg/dl for CRP, 0–240 µg/dL for D-dimer, 24–195 U/L for creatine kinase (CK), 0–25 U/L for creatine kinase-MB (CK-MB), 0–46.47 ng/mL for hs-Troponin I, 37.1–45.7 fL for RDW-SD and 1.26–3.35 10^3^/µL for lymphocytes.

### 2.4. Chest Computerized Tomography Imaging

Chest CT images were obtained from the hospital automation system registry. A multislice CT scanner (TOSHIBA Alexion 16 slice) was used for CCT imaging in the emergency room. Chest computed tomography (CCT) images of the study patients were interpreted by the radiology instructor. In CCT images, the presence of ground glass opacity (GGO), consolidation, air bronchogram, halosign, interlobular septal thickening and crazy paving pattern, as well as the number of involved lobes, were evaluated. The CCT images were grouped according to the findings as normal, mild, moderate or severe [5]. The CCT images were classified according to the findings, as follows: Normal: CCT images were normal; Mild: slight ground-glass opacity (GGO) in the periphery of the lung parenchyma, involvement of one or two lobes and less than half of each lobe; Moderate: bilateral and more than two-lobe involvement of the lungs, more than half of each lobe, with consolidation; Severe: presence of heavy consolidation, air bronchogram, halo sign, inter-lobular septal thickening and crazy paving pattern, along with multiple lobe involvement in the lungs.

### 2.5. Statistical Analysis

Analysis of the data collected in the study was performed using the Social Sciences 27 statistical software package (SPSS 27(IBM, New York, NY, USA)). The Kolmogorov–Smirnov test was used to determine whether continuous and discrete random variables were normally distributed. Descriptive statistics were given as mean ± standard deviation or as median (interquartile range) for continuous and discrete random variables, whereas categorical variables were given as the number of cases and their percentages (%). Categorical variables were assessed with the chi-squared test, parametric data with the Student’s t-test and non-parametric data with the Mann–Whitney U test and the Kruskal–Wallis H test. By utilizing receiver operating characteristic (ROC) analysis, the area under the curve (AUC) was used to calculate the cut-off value, specificity and sensitivity. To determine the statistical significance and assumptions, *p* < 0.05 with 95% confidence intervals was considered significant in all analyses. The power analysis was performed using G*Power version 3.1.9.7 (2020) for Windows 10 (Universitat Düsseldorf, Germany), referencing similar studies in the literature. With a power of 0.95 and a type 1 error rate of 0.05, the sample size was calculated as 116.

The patients were divided into two groups. The surviving patients were included in Group 1 (survivors), and the patients who died were included in Group 2 (non-survivors). The ECG, laboratory results and CCT findings of the two groups were compared.

## 3. Results

**Figure 1:**
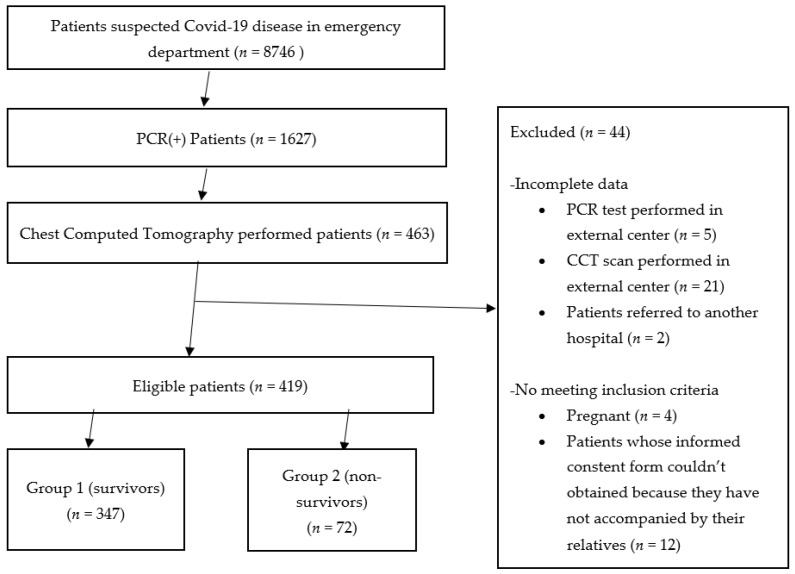
Patient flow chart.

The study form was completed for 463 patients. Fourty-four patients were excluded from the study because the PCR test (five patients) or the CCT scan (21 patients) were performed at an external center, while four patients were pregnant and two were referred to another hospital. Additionally, patients not accompanied by their relatives (12 patients) were excluded from the study because the informed consent form could not obtained from these patients. Of the 419 patients included in the study, 347 (83%) survived (Group 1) and 72 (17%) died in the hospital (Group 2) (Figure 1). The retrospective analysis of the results showed there were 139 (40%) women and 208 (60%) men in Group 1 and 28 (39%) women and 44 (61%) men in Group 2 (*p* = 0.895). The age of the patients was 51 ± 17 years and 64 ± 1 5 years in Groups 1 and 2, respectively (*p* = 0.001). There was a difference between the two groups in terms of their complaints when admitted to the ED. In Group 1, 16% of the patients had no complaints but had a history of contact with a COVID-19 patient. In Group 2, 69% of the patients admitted to the ED complained of dyspnea. When the diseases existing in their history were evaluated, it was observed that diabetes mellitus (DM), coronary artery disease (CAD), cancer and stroke were more common in the Group 2 patients. There was no difference between the groups in terms of hypertension (HT) and chronic obstructive pulmonary disease (COPD) diseases (Table 1, Figure 1).

When the vital signs measured at the time of admission to the emergency service were compared, the heart rate and respiratory rate were found to be higher, and the peripheral oxygen saturation (SpO_2_) and diastolic blood pressure (DBP) were found to be lower in the non-survivor patients compared to the survivor patients. There was no difference in the fever levels. In addition, QRS and QTc were measured as longer in the non-survivor patients (Table 2). There were no patients with OTc > 500 ms in either group. The QRS was > 120 ms in 4 (1%) of the survivor patients and 13 (18%) of the non-survivor patients.

White blood cell (WBC), neutrophil, neutrophil–lymphocyte ratio (NLR), lactate, D-dimer, fibrinogen, C-reactive protein (CRP), urea, creatinine, CK-MB and hs-Troponin I (hs-Tn I) levels were found to be higher and PCO_2_, BE, HCO_3_, lymphocyte (lym) and eosinophil (eos) levels were found to be lower in non-survivor patients.

There was a 32% increase in the WBC level, a 69% increase in the neutrophil level and a 28% decrease in the lym level in the non-survivor patients compared to the survivor patients. The median value of the eos level in non-survivor patients was 0.00 (10^3^/µL). 

The non-survivor patients had approximately a 3-fold increase in the D-dimer level, approximately a 5-fold increase in the CRP level and a 2.5-fold increase in the NLR compared to the survivor patients.

**Figure 2:**
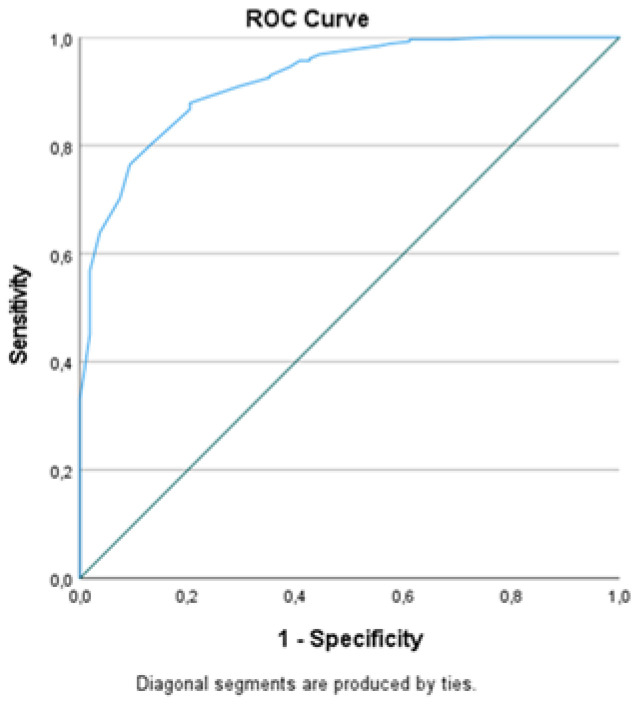
ROC analysis of SpO_2_ as mortality predictor.

ROC analysis was performed for the parameters in which there was a statistical difference between the survivor and non-survivor patients. Accordingly, the area under the curve (AUC) for SpO_2_, DBP, pCO_2_, BE, HCO_3_, hemoglobin (Hgb), lym and eos was found as higher than 0.600. The AUC of NLR, lactate, D-dimer, fibrinogen, CRP, urea, creatinine, CK-MB and hs-Tn I was calculated as less than 0.600. The highest AUC was calculated at the SpO_2_ level and the next highest at the eos level. When the cut-off value for SpO_2_ was determined as 90%, the sensitivity was 87% and the specificity was 80%. When the cut-off value for eos was 0.00 (10^3^/µL), the sensitivity and the specificity were 76% and 60%, respectively (Table 3, Figure 2). 

When the ECG findings were evaluated, atrial fibrillation, left bundle branch block, left ventricular hypertrophy and ST segment depression were more common in non-survivor patients compared to survivor patients (*p* = 0.001). 

**Figure 3:**
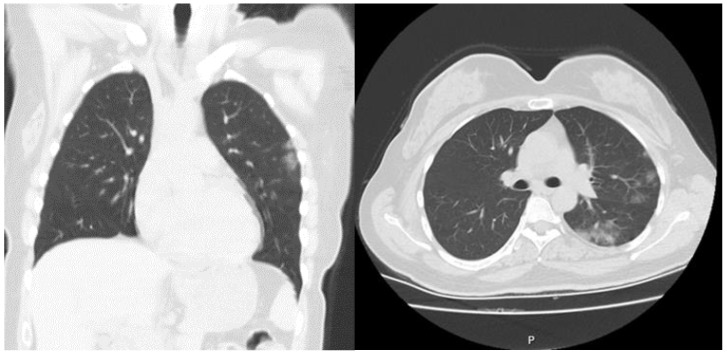
A 45-year-old female patient was admitted to the emergency department with complaint of cough started after 3 days of a diagnosis of COVID-19 disease. She had no chronical disease. Vital signs on admission were fever: 37 °C, pulse rate (PR): 90/min, respiratory rate (RR): 18/min, SpO_2_:97% and blood pressure (BP): 110/70 mmHg. Laboratory findings were: BE: 0.7 mmol/L, HCO_3_: 23.2 mmol/L, Lactat: 1.7 mmol/L, WBC: 6.23 (10^3^/µL), Hgb: 12.7 g/dL, RDWSD: 37.4 fL, lym:3.01 (10^3^/µL), eos: 0.14 (10^3^/µL), D-dimer: 162 µg/L, hs-TnI: 0 ng/mL. She was followed and treated at home. She started to work after a 2-week home quarantine.

**Figure 4:**
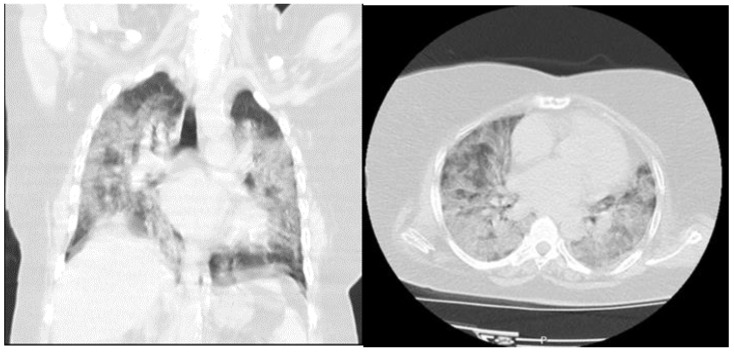
A 48-year-old female patient was admitted to the emergency department with complaint of dyspnea started after 5 days of a diagnosis of COVID-19 disease. She had no chronic disease. Vital signs on admission were fever: 36.8 °C, pulse rate (PR): 88/min, respiratory rate (RR): 32/min, SpO_2_: 80% and blood pressure (BP): 90/50 mmHg. Laboratory findings were: BE: 5.4 mmol/L, HCO_3_: 28 mmol/L, Lactat: 2.8 mmol/L, WBC: 7.57 (10^3^/µL), Hgb: 10.9 g/dL, RDWSD: 43.6 fL, lym: 2.12 (10^3^/µL), eos: 0.00 (10^3^/µL), D-dimer: 587 µg/L, hs-TnI: 4.07 ng/mL. She was hospitalized in the intensive care unit with the diagnosis of COVID-19 pneumonia. She died 7 days after admission.

When the CCT findings were evaluated, 79.2% of the non-survivor patients had severe findings, while 11.5% of the survivor patients had severe findings. The severity of lung involvement in CCT was compared with the SpO_2_ levels. Accordingly, the median value of SpO_2_ was 98% (IQR: 96–98%) in patients with normal lungs, 97% (IQR: 95–98%) for mild lung involvement, 94% (IQR: 92–96%) for moderate lung involvement and 84% (IQR: 74–88%) for severe lung involvement. There was a statistical difference between the groups according to admission to hospitalization units, and 89% of the non-survivor patients were admitted to the ICU. (Table 4, Figure 3 and Figure 4).

The Group 2 patients died after a median of 2 (IQR: 1–5) days from the day the PCR test was administered and a median of 6 (IQR: 3–11) days after admission to the emergency room.

## 4. Discussion

In this study, we prospectively included patients with suspected COVID-19 infection and retrospectively compared their clinical characteristics at admission to the ED, in two groups of survivor and non-survivor, and it was observed that DM, CAD, cancer and stroke were more common in the history of the non-survivor patients. In contrast, there was no difference between the survivor and non-survivor patients in terms of HT and COPD diseases. In addition, heart rate and respiratory rate were found to be higher in non-survivor patients, while SpO_2_ and DBP were found to be lower in the same patients. There was no difference between the survivor and non-survivor patients in terms of fever. When the ROC analysis of the vital findings was performed, the highest AUC was calculated at SpO_2_. The median value of SpO_2_ was 80% in the non-survivor patients and 95% in the survivor patients. In the studies of patients with COVID-19 pneumonia, a significant relationship was found between death and advanced age, male gender, SpO_2_, respiratory rate and cardiac troponin I. In addition, against all the variables, PaO_2_ ≥ 80 mmHg was determined to be the only factor associated with survival [1,6]. These results support our study’s findings.

The effects of SARS-CoV-2 on the heart are variable. The prognosis is worse when the heart is involved in the COVID-19 disease. The ECG is the primary preferred tool for assessing cardiac involvement, and the severity of COVID-19 and ECG findings were compared in many studies. In these studies, rhythm abnormalities, bundle branch blocks, ST segment abnormalities, ischemic T wave inversion, right ventricular overload and QT prolongation abnormalities were found to be associated with death or mechanical ventilation support [7,8,9]. Non-specific ECG findings reported in COVID-19 patients have been attributed to hypoxia, inflammatory damage and pulmonary embolism as a complication of COVID-19 and the drugs used in its treatment [3]. In our study, an ECG was obtained from all patients at the time of admission to the ED. Contrary to other studies, the patients in our study were diagnosed with COVID-19 in the ED and were not receiving any treatment when admitted to the ED. Atrial fibrillation, left bundle branch block, left ventricular hypertrophy and ST segment depression was observed more in the ECG analysis of non-survivor patients. Additionally, the QRS and QTc times were found to be longer in these patients. However, there was no patient with a QTc >500 ms in our study. The ratio of patients with a QRS >120 ms was 1% in survivors and 18% in non-survivors.

Laboratory studies are non-specific in the diagnosis of COVID-19. The most common laboratory findings in these patients are normal/low lymphocyte count and increased levels of CRP, D-dimer, lactate dehydrogenase, aminotransferase and ferritin. In contrast, the procalcitonin level is typically normal. As well as cardiac troponin I, N-terminal pro-brain natriuretic peptide and IL-6 levels were found to be significantly higher in the non-survivor patients than in the survivor patients [10,11,12,13]. In one study, it was reported that in-hospital mortality could be effectively predicted if there is a 4-fold increase in D-dimer level measured at hospital admission [14]. In another study, increased RDW (>14.5%) was found to be associated with an increased risk of mortality in patients of all ages. The mortality rate was calculated as 11% in patients with normal RDW and 31% in patients with high RDW [15]. In a study comparing blood eosinophil level and mortality rates in COVID-19 patients, it was shown that the mortality of patients increased as eosinopenia became worsened [16]. 

In our study, there was a 32% increase in the WBC level, a 69% increase in the neutrophil level and a 28% decrease in the lym level in the non-survivor patients compared to the patients who survived. Eosinopenia detected in non-survivor patients and the median value of the eos level was 0.00 (10^3^/µL). The RDW-SD level was found to be higher in the non-survivor patients. The non-survivor patients had an approximately 3-fold increase in D-dimer level, an approximately 5-fold increase in CRP level and a 2.5-fold increase in the NLR compared to the survivor patients. These findings suggest that inflammation was more severe in the non-survivor patients and indicate the presence of other pathogens together with SARS-CoV-2 in these patients. When the ROC analysis of parameters with a statistical difference between the survivor and non-survivor patients was performed, the AUC was found to be higher than 0.600 only for Hgb, lym and eos. The highest AUC was calculated at the eos level. When the cut-off value for eos was determined as 0.00 (10^3^/µL), the sensitivity was 76% and the specificity was 60%. On the basis of this finding, we concluded that the eosinophil level could be used as a prognostic marker.

In studies investigating the CCT scans of COVID-19 patients, disease progression was found to be correlated with an increase in the number, density and width of GGOs [4,17,18]. It has been reported that the number of involved lung segments and lobes, the frequency of consolidation, the crazy paving pattern and the air bronchogram are increased in more severe cases [5]. Additionally, a correlation has been found between the PaO_2_:FiO_2_ ratios, PCO_2_ and SpO_2_ levels and the lung involvement of the patients [19,20]. In one study, a weak correlation was found between the consolidation–fibrosis score and pCO_2_, while a moderate correlation was found between pO_2_ and the consolidation fibrosis score in the CT scan [21]. These studies have reported that the PaO_2_ and SaO_2_ levels can be prognostic markers as they predict the extent of the inflammation area in a CT scan.

In our study of patients with COVID-19, the CCT findings were grouped as normal, mild (GGO), moderate (consolidation) and severe (fibrosis). When the CCT findings were evaluated, 79.2% of the non-survivor patients had severe findings, while 11.5% of the survivor patients had severe findings. In our study, there was a difference between the survivor and non-survivor patients according to the pCO_2_, BE, HCO_3_ and lactate levels measured in a venous blood gas analysis. When compared to the survivor patients, the non-survivor patients had lower levels of pCO_2_, BE and HCO_3_ and higher lactate levels. When the ROC analysis of these parameters was performed, the AUC for pCO_2_, BE and HCO_3_ was found to be higher than 0.600. The AUC of lactate was found to be less than 0.600. However, the highest AUC was calculated at SpO_2_. When the cut-off value for SpO_2_ was determined as 90%, the sensitivity was 87% and the specificity was 80%. In addition, in our study, we compared the severity of lung involvement in the CCT and SpO_2_ levels. Accordingly, the median value of SpO_2_ was 98% (IQR: 96–98%) in patients with normal lungs, 97% (IQR: 95–98%) in mild lung involvement, 94% in moderate lung involvement (IQR: 92–96%) and 84% in severe lung involvement (IQR: 74–88%). These findings indicate that SpO_2_ can be an effective marker in predicting mortality and CCT findings. A study analyzing the targeted oxygen saturation of COVID-19 patients supports our results. In this study, it is recommended to monitor SpO_2_ with pulse-oximetry and keep SpO_2_ in the range of 92–96% (in patients that are normoxemic at pre-COVID baseline) in both inpatients and outpatients [22].

### 4.1. Limitations

Patients who were admitted to the ED were included in our study. Therefore, the laboratory parameters studied in the emergency department were compared. The main limitations of our study were that the mortality studies could not be conducted with more detailed laboratory parameters, and also that our study was conducted in a single center. Therefore, the number of patients in the study was limited.

### 4.2. Recommendations

In COVID-19 patients, SpO2, one of the vital signs measured in the emergency room, can be used as the first marker to show clinical severity before conducting laboratory and imaging studies. Patients with low SpO2 levels in the emergency department should be investigated immediately and followed closely. In order to reduce mortality in these patients, SpO2 should be kept at 92% and above. Pulse –oximetry records of outpatients should be monitored and target oxygen saturation information should be given to the patient. Laboratory results should be followed closely to determine prognosis in inpatients.

## 5. Conclusions

COVID-19 is a fatal disease whose mortality risk can be estimated when the clinical, laboratory and imaging studies of the patients are evaluated together in the ED. SpO_2_ that is measured before starting oxygen therapy, the eosinophil levels and the CT findings are all important predictors of mortality risk. Additionally, D-dimer, CRP, NLR, WBC, neutrophil and lymphocyte levels can be used to predict mortality. However, multi-centered studies are needed to evaluate these results.

## Figures and Tables

**Table 1:** Patients’ complaints and chronic diseases.

Groups	Group 1 (Survivor) *n* (%)	Group 2 (Non-Survivor) *n* (%)	*p* Value
**Complaints of Patients in Emergency Service**			
No complaint (a history of contact with a COVID-19 patient in the anamnesis)	57 (16)	1 (1)	0.001
Fever	40 (12)	2 (3)	
Cough	37 (10)	7 (10)	
Dyspnea	99 (29)	50 (69)	
Joint Pain	32 (9)	2 (3)	
Weakness	64 (18)	7 (10)	
Diarrhea	7 (2)	2 (3)	
Nausea andVomiting	9 (3)	1 (1)	
Anosmia	2 (1)	0	
Total	347	72	
**Cronical Diseases**			
Diabetes Mellitus	25 (7.2)	11 (15.3)	0.036
Hypertension	87 (25)	16 (22.2)	0.655
Coronary Artery Disease	18 (5.2)	13 (18.1)	0.001
Chronic Obstructive Pulmonary Disease	23 (6.6)	5 (6.9)	1.000
Renal Failure	3 (0.9)	1 (1.4)	0.531
Cancer	0	2 (2.8)	0.029
Stroke	2 (0.6)	5 (6.9)	0.002

**Table 2:** Comparison of the clinical and laboratory results of survivor and non-survivor patients.

Groups	Group 1 (Survivor)		Group 2 (Non-Survivor)		*p* Value
	Median	IQR	Median	IQR	
**Vital Signs**					
Fever (°C)	36.7	36.2–37.4	36.7	36.5–38	0.084
PR (beat per minute)	88	78–100	100	88–118	0.001
RR (breaths per minute)	22	18–27	35	30–39	0.001
SBP (mmHg)	130	110–140	120	100–140	0.177
DBP (mmHg)	80	70–80	70	60–80	0.009
SpO_2_ (%)	95	92–97	80	69–88	0.001
**Electrocardiogram**					
ECG rate (beat per minute)	85	75–95	96	81–102	0.008
QRS	90	84–96	116	92–150	0.001
QTc (ms)	404	392–416	443	414–451	0.001
**Hemogram**					
Wbc (10^3^/µL)	6.12	4.88–7.92	8.08	5.46–11.18	0.001
Hgb (g/dL)	13.6	12.4–14.8	12.9	11.4–14.2	0.002
Hct (%)	40.2	37.5–43.1	38.7	34.6–42.6	0.037
MCV (fL)	83.5	80.7–86.3	85.4	81.5–90.9	0.003
RDW-SD (fL)	39.7	37.2–42.3	43.2	40.5–47.9	0.001
Plt (10^3^/µL)	207	170–252	206	145–249	0.366
Lym (10^3^/µL)	1.47	1.09–2.03	1.06	0.80–1.38	0.001
Neu (10^3^/µL)	3.88	2.71–5.54	6.57	3.83–9.33	0.001
Eos (10^3^/µL)	0.02	0.01–0.07	0.00	0.00–0.01	0.001
Baso (10^3^/µL)	0.01	0.001–0.03	0.01	0.00–0.02	0.106
Mono (10^3^/µL)	0.51	0.39–0.68	0.44	0.31–0.61	0.024
NLR	2.56	1.65–4.34	6.45	3.96–9.26	0.001
**Venous Blood Gases**					
pH	7.40	7.37–7.42	7.38	7.34–7.42	0.036
PCO_2_ (mmHg)	43.4	38.5–48.1	38.8	34.1–47.2	0.004
PO_2_(mmHg)	29	25–37	29.4	21.9–36	0.517
BE (mmol/L)	1.9	0.2–3.6	−0.5	−4.4–1.9	0.001
HCO_3_ (mmol/L)	25.2	23.8–27	22.9	19.9–25.1	0.001
Lactat (mmol/L)	1.5	1.1–1.8	2.2	1.4–4.3	0.001
**Laboratory Results**					
D-Dimer µg/L)	177	105–285	482	215–791	0.001
Fibrinogen (mg/dL)	421	314–554	587	462–693	0.001
CRP (mg/dL)	2.9	0.5–8.4	13.8	9.2–16.5	0.001
Urea (mg/dL)	27	21–37	53	34–76	0.001
Creatinin (mg/dL)	0.85	0.75–1.04	1.03	0.83–1.69	0.001
CK (U/L)	82	52–151	97	44–220	0.266
CK-MB (U/L)	16.5	13–22.8	21.6	15.5–29.6	0.001
hs-Troponin I (ng/mL)	0	0–5.08	15.15	4.74–40.27	0.001

IQR: interquartile range, PR: Pulse Rate, RR: Respiratory Rate, SBP: Systolic Blood Pressure, DBP: Diastolic Blood Pressure, SpO_2_: Peripheral Oxygen Saturation, ECG: Electrocardiogram, Wbc: White Blood Cell, Hgb: Hemoglobin, Hct: Hematocrit, MCV: Mean Corpuscular Volume, RDW-SD: Red Cell Distribution Width-Standard Deviation, Plt: Platelet, Lym: Lymphocyte, Neu: Neutrophil, Eos: Eosinophil, Baso: Basophil, Mono: Monocyte, NLR: Neutrophil/Lymphocyte, BE: Base Excess, CRP C-Reactive Protein, CK: Creatine kinase, CK-MB: Creatine kinase-MB.

**Table 3:** ROC analysis of parameters that have statistical differences between survivor and non-survivor patients.

Test Result Variable (s)	Area under Curve (AUC)	Asymptotic 95% Confidence Interval	
		Lower Bound	Upper Bound
RR	0.044	0.023	0.065
SpO_2_	0.921	0.885	0.957
DBP	0.622	0.531	0.712
PR	0.375	0.248	0.503
ECG rate	0.332	0.215	0.448
ECG QTC	0.347	0.226	0.467
WBC	0.357	0.281	0.434
HGB	0.616	0.545	0.687
HCT	0.578	0.499	0.657
MCV	0.389	0.310	0.468
RDW-SD	0.242	0.180	0.304
NEU	0.285	0.217	0.352
LYM	0.695	0.629	0.761
EOS	0.710	0.644	0.776
MONO	0.585	0.508	0.662
NLR	0.214	0.158	0.270
pH	0.586	0.499	0.673
PCO_2_	0.617	0.532	0.703
BE	0.687	0.602	0.773
HCO_3_	0.735	0.661	0.809
Lactat	0.282	0.197	0.366
D-Dimer	0.228	0.171	0.286
Fibrinogen	0.264	0.201	0.328
CRP	0.154	0.112	0.196
Urea	0.194	0.138	0.251
Creatinin	0.319	0.245	0.394
CK-MB	0.371	0.295	0.448
hs-Troponin I	0.182	0.126	0.239

PR: Pulse Rate, RR: Respiratory Rate, SBP: Systolic Blood Pressure, DBP: Diastolic Blood Pressure, SpO_2_: Peripheral Oxygen Saturation, ECG: Electrocardiogram, Wbc: White Blood Cell, Hgb: Hemoglobin, Hct: Hematocrit, MCV: Mean Corpuscular Volume, RDW-SD: Red Cell Distribution Width-Standard Deviation, Plt: Platelet, Lym: Lymphocyte, Neu: Neutrophil, Eos: Eosinophil, Baso: Basophil, Mono: Monocyte, NLR: Neutrophil/Lymphocyte, BE: Base Excess, CRP C-Reactive Protein, CK: Creatine kinase, CK-MB: Creatine kinase-MB.

**Table 4:** Comparison of survivor and non-survivor patients according to ECG and CT findings and follow-up and treatments of all patients after the emergency service.

Groups	Group 1 (Survivor) *n* = 347 (%)	Group 2 (Non-Survivor) *n* = 72 (%)
**ECG Findings**		
Atrial fibrillation	5 (1.4)	3 (4.2)
RBBB	11 (3.2)	2 (2.8)
ST segment depression	2 (0.6)	2 (2.8)
T dalga inversion	11 (3.2)	1 (1.4)
LBBB	3(0.9)	4 (5.6)
LVH	4 (1.2)	2 (2.8)
S1Q3T3	14 (4)	2 (2.8)
**Chest Computed Tomography**		
Normal	74 (21.3)	0
Mild	95 (27.4)	0
Moderate	138 (39.8)	15 (20.8)
Severe	40 (11.5)	57 (79.2)
**Follow-up and Treatments**		
Patients followed and treated at home	139 (40)	0
Service	186 (54)	8 (11)
Intensive Care Unit	22 (6)	64 (89)

RBBB: Right Bundle Branch Block, LBBB: Left Bundle Branch Block, LVH: Left Ventricular Hypertrophy.
